# Identification and validation of shared key genes between Parkinson’s disease and erectile dysfunction: a bioinformatics approach

**DOI:** 10.1186/s41065-025-00614-1

**Published:** 2025-11-25

**Authors:** Yincheng Fan, Guangqian Gao, Yurong Xiang, Haibo Zhang, Shuhua He, Anyang Wei

**Affiliations:** Department of Urology, Nanfang Hospital, No. 1838, Guangzhou Avenue North, Baiyun District, Guangzhou, 510515 Guangdong Province China

**Keywords:** Erectile dysfunction, Parkinson’s disease, Machine learning, PIK3R6, SHOX2, Immune infiltration

## Abstract

**Background:**

Erectile dysfunction (ED) and Parkinson’s disease (PD) are prevalent conditions that considerably impair patients’ quality of life. Emerging evidence suggests a potential relationship between ED and PD, possibly mediated by shared biological mechanisms. This research seeks to examine shared transcriptomic alterations and the underlying biological pathways associated with ED and PD.

**Methods:**

Gene expression profiles related to ED and PD were derived from the Gene Expression Omnibus database, specifically the GSE2457 and GSE7621 datasets. Differentially expressed genes (DEGs) between patients and controls were identified through differential expression analysis. Functional enrichment analyses, including Kyoto Encyclopedia of Genes and Genomes (KEGG) pathway and Gene Ontology analyses, were carried out to uncover the biological roles of the identified DEGs. To refine and validate potential key genes, machine learning algorithms, such as support vector machine-recursive feature elimination and LASSO regression, were employed. Immune infiltration analysis was carried out to examine potential immune responses related to the identified genes. Additionally, miRNA-gene and protein–protein interaction networks were established. Finally, the reliability of the selected genes was validated through external and experimental verification.

**Results:**

In total, 25 overlapping DEGs were identified between ED and PD. Functional enrichment analysis demonstrated that these DEGs were involved in such biological processes as redox homeostasis and neuronal cell body function. KEGG pathway analysis indicated significant enrichment in pathways such as adrenergic signaling, cGMP-PKG signaling. Machine learning algorithms further refined the candidate genes, with *SHOX2* and *PIK3R6* demonstrating strong diagnostic potential. Immune infiltration analysis demonstrated correlations between the gene expression levels and various immune cell types. The constructed miRNA-gene regulatory networks revealed possible post-transcriptional regulatory mechanisms that modulated the expression of these genes. Finally, the diagnostic performance of these genes was verified in external datasets, with their performance further confirmed by ROC analysis and experimental verification.

**Conclusion:**

This study identified the shared biological target between ED and PD through bioinformatics analyses. The key genes *SHOX2* and *PIK3R6* may serve as potential biomarkers. These results may offer new insights into the molecular mechanisms linking ED and PD.

**Supplementary Information:**

The online version contains supplementary material available at 10.1186/s41065-025-00614-1.

## Background

Erectile dysfunction (ED) refers to the consistent inability to attain or maintain an erection sufficient for satisfactory sexual performance [1]. As a prevalent but multifactorial male sexual dysfunction, ED involves alterations in various components of the erectile response, such as psychological, organic, and relational factors. Both nonendocrine (iatrogenic, vasculogenic, and neurogenic) and endocrine pathways contribute to its pathogenesis [2]. Endocrine disorders implicated in ED include thyroid dysfunction, hyperprolactinemia, diabetes mellitus (DM), hypogonadism, and impaired glucose tolerance [3].

Neurogenic ED is a major cause of non-endocrine disorders [4]. It is caused by impaired nerve signaling to the corpora cavernosa [5]. Lesions in upper motor neurons (above spinal nerve T10) typically disrupt the erection control mediated by the central nervous system (CNS) without local penile changes. In contrast, sacral lesions (S2–S4), which mediate reflexogenic erections, lead to structural and functional alterations owing to the decreased innervation [6]. Parkinson’s disease (PD) is one of the multiple etiologies of neurogenic ED. PD refers to a neurodegenerative disorder primarily marked by a distinctive motor phenotype (Parkinsonism) [7], including tremor, bradykinesia, rigidity, and altered gait and postural reflexes [8]. Individuals with PD frequently experience other non-motor symptoms, including neuropsychiatric disorders, cognitive decline, sleep disturbances, sensory deficits, and autonomic dysfunction, which may be more disabling than motor symptoms [9]. ED is a prevalent non-motor symptom in men with PD and is considered a manifestation of autonomic nervous system dysfunction [10]. Moreover, ED is considered a risk factor contributing to the onset of PD [11, 12]. The bidirectional relationship between ED and PD has been examined in a growing body of literature. A population-based longitudinal study involving 15,765 patients found that individuals with ED had a 1.52-fold greater risk of PD in comparison to those without ED, after controlling for comorbidities and age [13]. The risk of PD was about 2.5 times higher among individuals with both ED and DM compared with those without either condition. In addition, in comparison to healthy controls, impaired sexual function is more prevalent among individuals with PD [14]. Although clinical and epidemiological evidence demonstrates a close association between PD and ED, the shared molecular features, particularly those related to gene regulatory mechanisms, remain poorly understood.

ED has been proposed as an early indicator for identifying individuals at increased risk of PD [15]. With recent advances in bioinformatics and sequencing technologies, it has become feasible to investigate the shared pathogenic mechanisms underlying disease-disease interactions at the genetic level [16, 17]. This research aimed to uncover the biological pathways linking ED and PD, thereby offering novel insights into early diagnosis and treatment.

## Methods

### Sample sources

All experimental data utilized in this study were obtained from the Gene Expression Omnibus (GEO) (https://www.ncbi.nlm.nih.gov/geo/). For ED analysis, the GSE2457 dataset was selected. This dataset comprised 10 rat samples, including 5 healthy controls and 5 ED [18]. Homologous genes between humans and mice were identified and mapped to human gene symbols for subsequent analysis. For PD analysis, the GSE7621 dataset was utilized. This dataset consisted of 25 human samples, including 16 PD patients and 9 healthy controls [19]. Additionally, two external validation datasets were employed. The validation dataset for ED was GSE10804, containing gene expression profiles (GEPs) from human endothelial cells isolated from 5 corpora cavernosa, 4 coronary arteries, and 3 umbilical veins [20]. In this study, the 5 samples derived from the corpus cavernosum (the primary effector organ of erection) were designated as the ED-related case group, while the 3 samples from umbilical vein endothelial cells (HUVEC), a standard model for ‘normal’ endothelium, served as the control group. The validation dataset for PD was GSE49036, consisting of 20 PD samples and 8 controls derived from human samples [21] (Table 1).

**Table 1:** Various datasets and introductions

Dataset	Species origin	Tissue	Sample distribution
GSE2457	Rat	Penile Tissues	5 controls and 5 ED
GSE7621	Homo sapiens	Human brain substantia nigra	9 control and 16 PD
GSE10804	Homo sapiens	Corpus cavernosum, coronary artery, coronary artery	5 corpora cavernosa, 4 coronary arteries, and 3 umbilical veins
GSE49036	Homo sapiens	human brain substantia nigra	8 controls and 20 PD

### Differential expression analysis (DEA)

The series matrix files of GSE2457 and GSE7621 were obtained from the GEO database. Quality control and normalization for the raw gene expression data were carried out prior to analysis. Subsequently, probe IDs were mapped to their corresponding gene symbols, and all expression values were transformed using a log2 scale. Differentially expressed genes (DEGs) between disease groups (ED or PD) and healthy controls were selected via the limma package in R (version 4.4.1). Differentially expressed genes were identified with a threshold of adjusted p-value < 0.05 and |log2(FC)| >0.5, using the Benjamini-Hochberg method to control the false discovery rate (FDR). The intersection analysis was carried out to obtain overlapping DEGs between the two datasets (GSE2457 and GSE7621). Visualization was conducted using the ggplot2 package to generate volcano plots illustrating the distribution of DEGs, and Venn diagrams were constructed to illustrate the overlap of DEGs between the two datasets.

### Functional enrichment analysis (FEA)

FEA was conducted to elucidate the biological roles of the identified DEGs. Gene Ontology (GO) enrichment analysis, covering molecular functions, cellular components, and biological processes, and pathway enrichment analyses (Kyoto Encyclopedia of Genes and Genomes [KEGG] and WikiPathways) were conducted using the Database for Annotation, Visualization, and Integrated Discovery (https://davidbioinformatics.nih.gov/home.jsp). Genes were considered significantly enriched based on the following criteria: gene count more than 2, EASE score under 1, and p-value less than 0.05. The results were presented as bubble plots and bar graphs via the “barplot” and “dotplot” functions from the “ggplot2” package.

### Leveraging machine learning (ML) for advanced gene filtering

The Least Absolute Shrinkage and Selection Operator (LASSO) regression was applied to the GSE2457 (ED) and GSE7621 (PD) datasets for feature selection using the “glmnet” and “magrittr” packages. By introducing an L1 regularization penalty, LASSO regression effectively reduced model overfitting and minimized interference from irrelevant genes, thereby enhancing the robustness of gene selection. The research workflow comprised: (i) standardization of the raw expression data; (ii) determination of optimal regularization parameter λ via ten-fold cross validation, followed by the identification of genes with non-zero regression coefficients (*p* < 0.05); (iii) secondary feature selection using the Support Vector Machine Recursive Feature Elimination (SVM-RFE) method via the rfe function, to obtain optimal feature subsets; (iv) integration of LASSO and SVM-RFE results from both datasets to identify shared feature genes between ED and PD through Venn diagram intersection analysis.

The diagnostic accuracy of the ML model was assessed through the receiver operating characteristic (ROC) analysis. For each gene, ROC curves were plotted, and the area under the curve (AUC) was calculated to quantify their ability to distinguish between disease and control samples. AUC values ranged from 0.5 to 1.0, with greater values suggesting stronger discriminative power. In addition, boxplots were employed to visualize the differential expression of these genes, revealing significant differences between disease and control groups (*p* < 0.05).

### Immune infiltration analysis

CIBERSORT stands as a widely employed computational method for quantitatively estimating the cellular composition of bulk tissue GEPs, particularly the abundance of immune cell subsets [22]. In this research, the CIBERSORT deconvolution algorithm was employed on the GSE2457 and GSE2461 datasets to estimate immune cell infiltration in disease and control samples according to gene expression signatures. The analytical workflow comprised: (i) implementation of the CIBERSORT algorithm in the R, using the LM22 signature matrix (containing GEPs of 22 immune cell subtypes) as the reference; (ii) execution of 1,000 permutations (PERM = 1000) and selection of significantly differential immune cell subtypes according to a statistical threshold of *p* < 0.05; (iii) removal of cell types with zero relative abundance; and (iv) visualization of immune cell composition through stacked bar plots and intergroup comparisons via boxplots, both generated via the ggplot2 package. Pearson correlation analysis was performed between the key genes filtered by ML and the levels of immune cell infiltration separately in the ED and PD datasets. Then, a correlation heatmap was plotted to depict the relationships between key genes and types of immune cells, providing a visual representation of their interactions across both disease contexts.

Significant differences were found in immune microenvironment features between disease and control groups. These findings may offer insights into disease-related immunoregulatory mechanisms.

### PPI and GeneMANIA network analysis

Given the complexity of biomolecular interactions, core hub genes were identified using the CytoHubba plugin in Cytoscape. To mitigate the potential bias inherent in any single topological metric, five distinct algorithms (MCC, Cluster coefficient, MNC, EPC, and BottleNeck) were applied and run independently. The top 10 genes from each algorithm were selected. These selected genes were intersected to identify high-confidence core hub genes supported by multiple methods [23]. Furthermore, to elucidate key post-transcriptional regulatory mechanisms, miRNA-gene regulatory networks for the candidate hub genes (*SHOX2* and *PIK3R6*) were constructed. The predictions were performed on the GeneMANIA online analysis platform (https://genemania.org/). The platform integrates interactions from multiple authoritative databases, including large, public, and available biological databases such as GEO, BioGRID, and IRefindex. The resulting interaction networks were visualized using Cytoscape software, where nodes represented genes or miRNAs and edges represented regulatory relationships between them [24].

### External validation of screened genes

To validate the screened genes, differential expression analysis (DEA) was performed using two additional datasets from the GEO database. For the ED group, given the lack of an independent transcriptomic dataset of DMED in rats, we employed the GSE10804 dataset. This dataset profiles human cavernosal endothelial cells. For the PD group, the GSE49036 dataset derived from human substantia nigra samples was used as the validation set. Subsequently, ROC curve analysis was performed on both datasets to appraise the diagnostic accuracy of the candidate genes.

### Experimental validation

All animal experimental protocols obtained approval from the Ethics Committee of the Laboratory Animal Research Center, Nanfang Hospital, Southern Medical University. For the ED group, 16 male Sprague-Dawley (S-D) rats, aged six to eight weeks and weighing 180–220 g, were derived from the Animal Experiment Center of Southern Medical University and acclimated for 7 days before experimentation. DM was induced via a single intraperitoneal injection of streptozotocin (STZ) at 70 mg/kg, freshly dissolved in 0.1 mmol/L sodium citrate buffer (pH 4.2) to a final concentration of 12 mg/mL. Rats received 1 mL of STZ solution per 200 g body weight. Four S-D rats in the controls were administered citrate phosphate buffer alone. The success of DM modeling was confirmed by a random blood glucose level ≥ 16.7mmol/L. After confirmation, diabetic rats were maintained for 8 weeks before erectile function was assessed via apomorphine testing. After 8 weeks, all rats with DM survived, and those exhibiting ED were designated as DMED rat models. In terms of the PD group, after one week of adaptation, SD rats were anesthetized intraperitoneally and fixed on a stereotaxic device. After cutting open the scalp, a cotton swab was used to gently wipe the surface of the skull to expose the anterior and posterior fontanelle points. The stereotaxic device was adjusted to align the heights of the anterior and posterior fontanelle points. With the fontanelle point as the origin, the coordinates of the injection site were located. Then, a skull drill was used to drill a hole along the injection coordinates to ensure that the microinjector did not touch the hole wall during needle insertion. A microsyringe was used to aspirate 6-OHDA. It was slowly inserted into the injection site, and 6-OHDA was injected into the target brain area at a rate of 0.5ul/min, with a dosage of 4 µl. The number of times of rotational behaviors in animals was observed. A Parkinson’s disease model was generally considered successful if over 7 rotational behaviors per minute were observed.

The mRNA expression levels of DEGs between the control group and experimental group were measured via the quantitative real-time polymerase chain reaction (qRT-PCR). Total RNA was obtained from samples of controls and experimental rats via the FastPure Complex Tissue Total RNA Isolation Kit (Cat: RC113-01, Vazyme, China). The miRNA 1 st Strand cDNA Synthesis Kit (AG11717, Accurate Biology, China) was employed to synthesize complementary DNA (cDNA) from the extracted RNA samples. Subsequently, the SYBR Green Pro Taq HS Pre-mixed qPCR Kit (AG11713, Accurate Biology, China) was applied to carry out qPCR. The mRNA gene levels were presented as mean ± standard deviation. One-way analysis of variance (ANOVA) was used to compare results, and data were visualized using GraphPad Software (San Diego, California, USA). Primer sequences are listed in Table 2.

**Table 2:** Gene primer sequence

Gene	Primer type	Sequence/Target sequence
β-tublin	Forward	5′- GATCA-AGATCATTGCTCCTCCTG − 3′
	Reverse	5′- AGGGTGTAAAACGCAG-CTCA − 3′
SHOX2	Forward	5′- GGGCTGCTAGCCAGTTTGAA − 3′
	Reverse	5′- ACGTTGCAATGACTATCCTGC − 3′
PIK3R6	Forward	5′- GGTGGACAGCTGAGAACAATC − 3′
	Reverse	5′- GCACAGCCTGTACACTCCTC − 3′

### Statistical analysis

All results were represented by the mean ± standard error (SE). ANOVA was utilized to conduct comparisons among groups. R software (version 4.4.1) and GraphPad Prism 8.4.3 were employed to carry out all statistical analyses. A p-value < 0.05 was regarded as statistically significant.

## Results

### Screening of DEGs

**Fig. 1:**
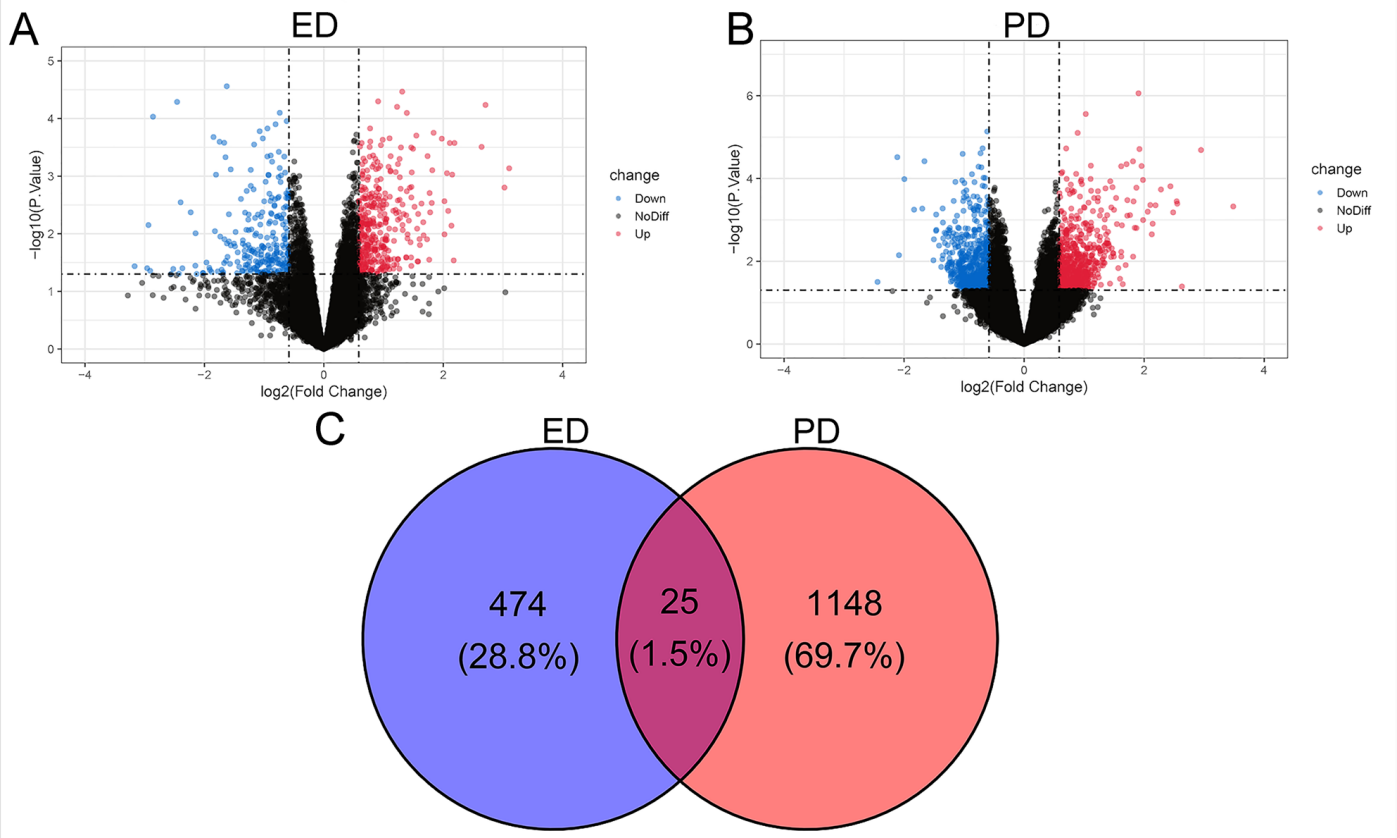
Identification of common DEGs in ED and PD. (**A**, **B**) Volcano plots displaying DEGs in the GSE2457 (ED) and GSE7621 (PD) datasets. (**C**) Venn diagram illustrating the overlap of DEGs between ED and PD

To identify genes potentially linked to both ED and PD, 1,672 DEGs were initially obtained from the GEO datasets GSE2457 and GSE7621 using a threshold of *p* < 0.05. Volcano plots of the DEGs are illustrated in Fig. 1A and B. By intersecting the two datasets, the shared genes between ED and PD were obtained. Among them, 25 overlapping genes were identified as significant androgen-sensitive related genes (ASRGs) (Fig. 1C). These genes may be valuable candidates for further investigation and offer insights into the underlying mechanisms of the two diseases.

### Functional enrichment analysis

**Fig. 2:**
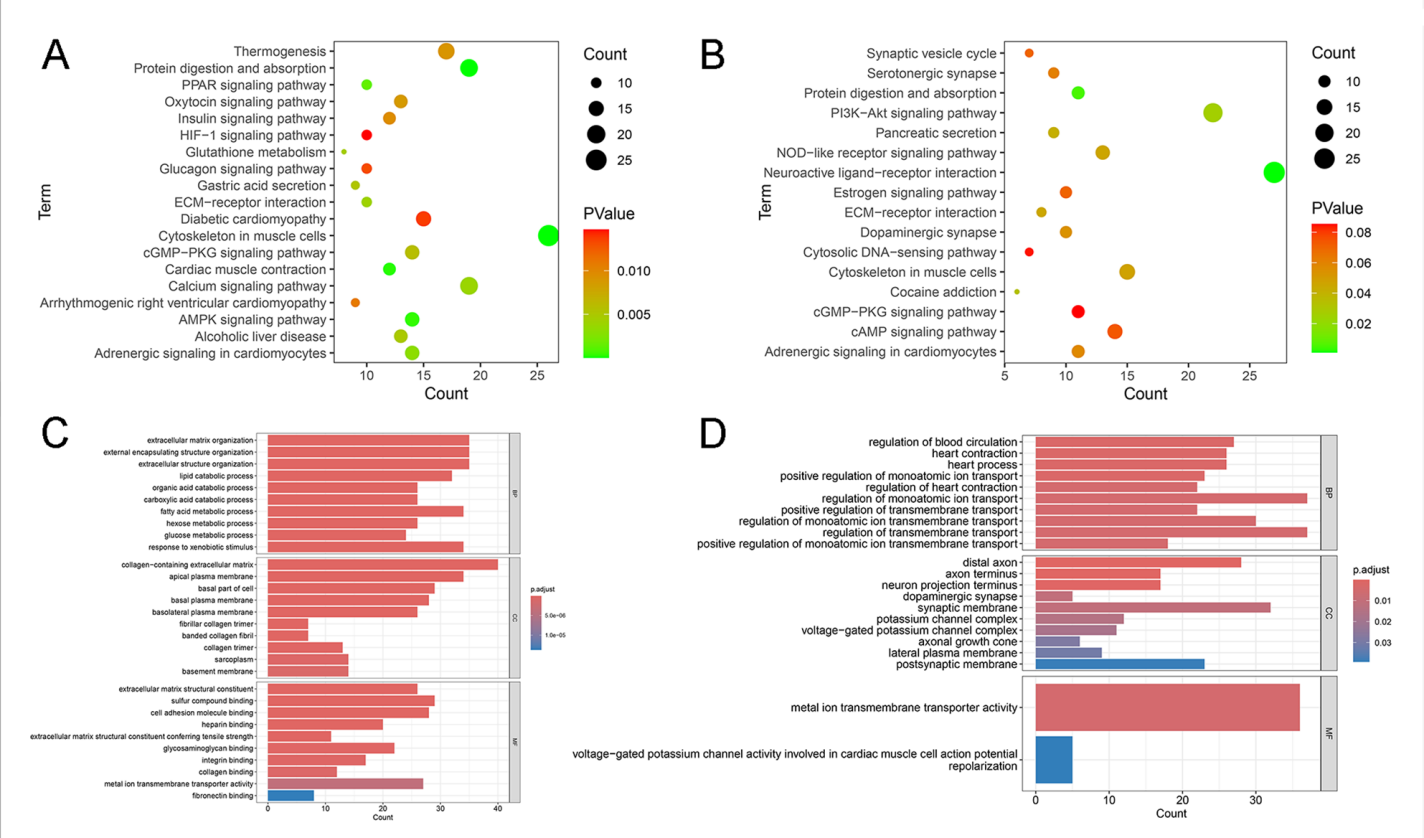
Functional enrichment analysis of DEGs in ED and PD. (**A**, **B**) KEGG analysis of DEGs from GSE2457 (ED) and GSE7621 (PD). (**C**, **D**) GO analysis of DEGs from GSE2457 and GSE7621

FEA was performed to investigate the potential biological functions of the DEGs and to examine the molecular mechanisms linking ED and PD. KEGG and GO analyses were conducted for the DEGs from the GSE2457 and GSE7621 datasets, respectively. As illustrated in Figs. 2(A) and 2(B), KEGG analysis demonstrated that these genes were mainly involved in adrenergic signaling cascades, the *cGMP-PKG* signaling pathways, cytoskeleton regulation, ECM-receptor interaction, and protein digestion and assimilation. As illustrated in Figs. 2(C) and 2(D), GO analysis demonstrated that these genes were primarily enriched in such pathways as metal ion transmembrane transporter activity and modulation of transmembrane transport. Subsequently, enrichment analysis was conducted on the 25 ASRGs: *TNMD*,* PIM3*,* BCAT1*,* FOXS1*,* NUPR1*,* HK2*,* ZIC1*,* RCAN2*,* FABP7*,* COL5A3*,* LRIT1*,* KCND3*,* PDLIM7*,* SHOX2*,* PIK3R6*,* GJB2*,* CPA1*,* ASIC2*,* RET*,* GPD1*,* SDS*,* SLC8A1*,* S100A4*,* NOMO3*,* NR4A2*. We also performed independent functional enrichment analysis on the 25 overlapping ASRGs. The KEGG analysis revealed that they were primarily enriched in virus-related pathways such as Kaposi sarcoma-associated herpesvirus infection and Human immunodeficiency virus 1 infection (Supplementary Fig. 1). However, the direct relevance of these pathways to the comorbidity of ED and PD is unclear and is biologically difficult to interpret. GO analysis revealed similarly dispersed and unfocused biological processes (Supplementary Fig. 1).

### ML–Based gene selection

To identify informative features within the gene expression data, two ML algorithms, namely LASSO and SVM-RFE, were applied for feature selection and dimensionality reduction.

**Fig. 3:**
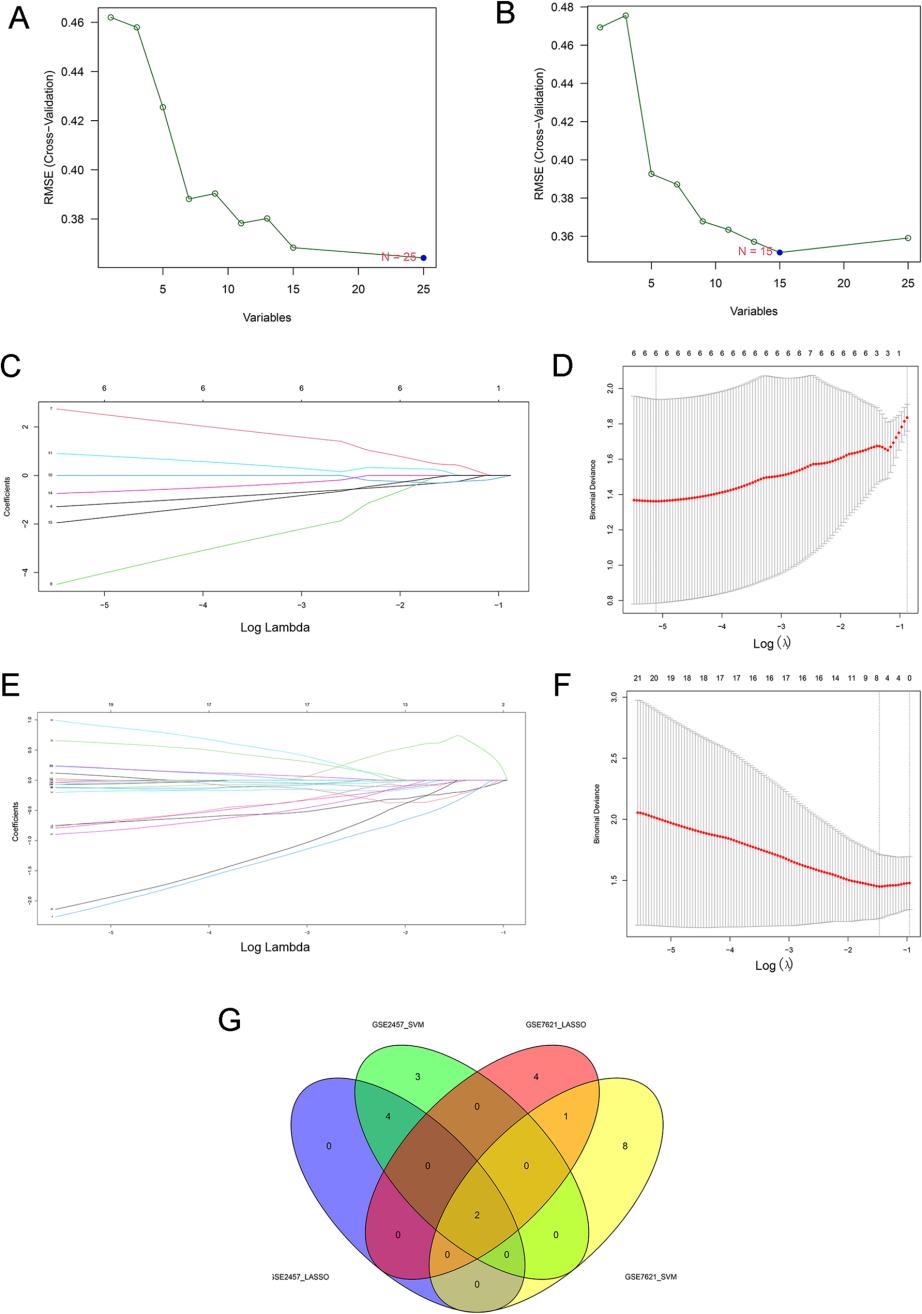
Machine learning for screening key genes. (**A**, **B**) Feature ranking using SVM-RFE, where the least important features were iteratively removed until an optimal subset was retained. (**C**, **E**) Gene selection using the LASSO algorithm based on the GSE2457 and GSE7621 datasets, respectively. (**D**, **F**) Gene selection using cross-validation. (**G**) Overlapping genes identified by both LASSO and SVM-RFE

SVM-RFE, which integrates SVM and RFE algorithms, was applied to the GSE2457 and GSE7621 datasets. Weight vectors generated during SVM training were utilized to rank genes by importance, and those with the lowest ranks were iteratively eliminated, resulting in a descending order of candidate genes (Fig. 3A and B).

In the GSE2457 dataset, six key genes were identified using the LASSO method and cross-validation (Fig. 3C and D). Similarly, in the GSE7621 dataset, these algorithms selected two key genes (Fig. 3E and F). The gene screening results of the four ML datasets have been presented in Table 3.

**Table 3:** ML gene screening results

GSE2457_lasso	Foxs1 Zic1 Rcan2 Lrit1 Shox2 Pik3r6
GSE2457_svm	Foxs1 Zic1 Rcan2 Lrit1 Foxs1 Gpd1 Zic1 Shox2 Pik3r6
GSE7621_lasso	Vn1r2 Banf1 Loc100133130 Hoxd11 Htr1f Shox2 Pik3r6
GSE7621_svm	Pdlim7 Kcnd3 Banf1 Slc18a2 Rbm3 Htr1F Ak05765 Ddx11 Ascl2 Shox2 Pik3r6

Ultimately, by intersecting the results of LASSO regression and SVM-RFE across the four datasets, two key genes, *SHOX2* and *PIK3R6*, were selected (Fig. 3G). These genes exhibited significant differential expression, providing valuable candidates for further investigation.

**Fig. 4:**
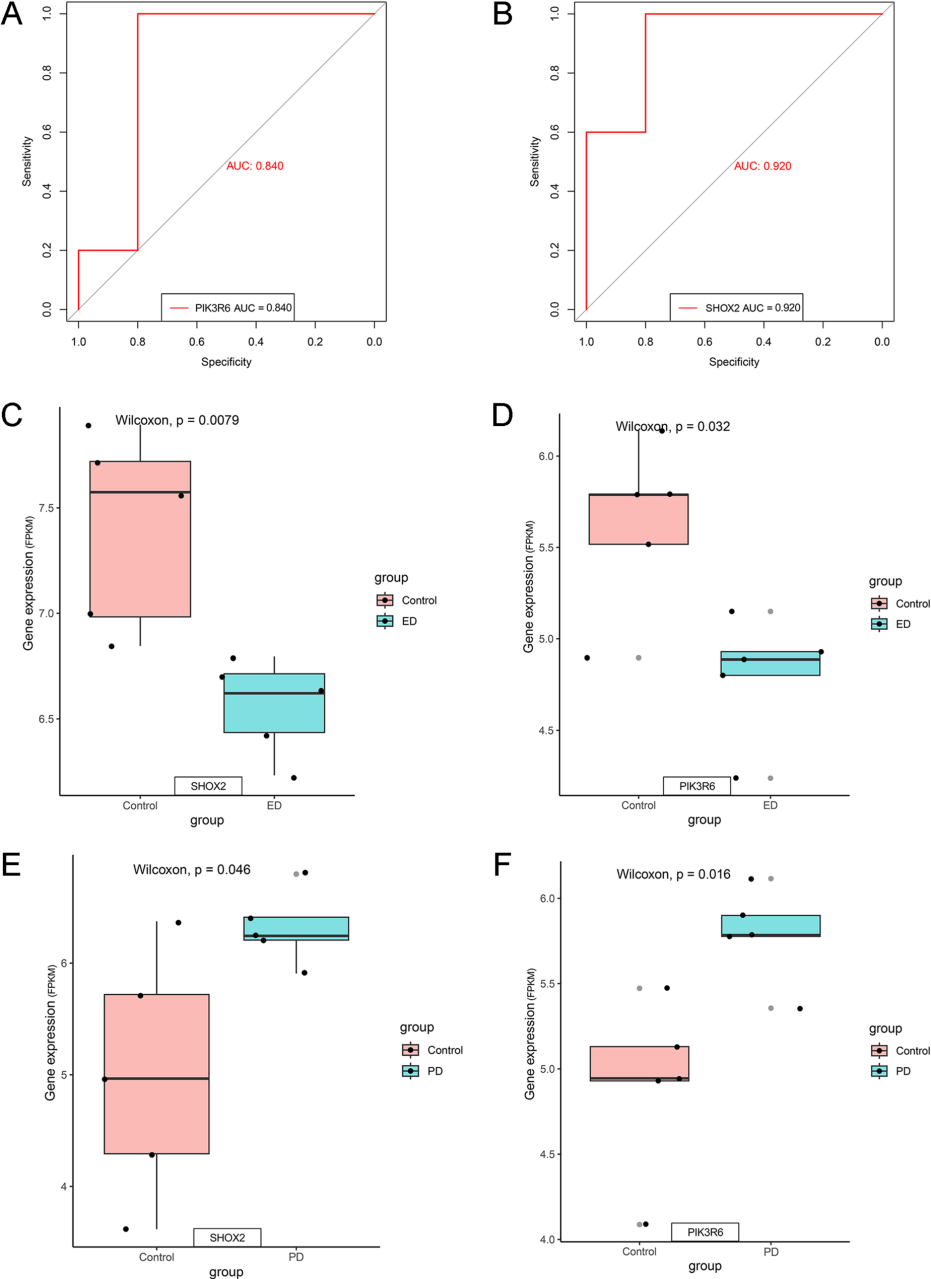
ROC curves and expression boxplots. (**A**) ROC curve of *PIK3R6*. (**B**) ROC curve of *SHOX2*. (**C**, **D**) Expression boxplots of *PIK3R6* and *SHOX2* in the ED dataset. (**E**, **F**) Expression boxplots of *PIK3R6* and *SHOX2* in the PD dataset

To evaluate the screened DEGs, ROC curves were plotted. The AUC was 0.840 for *PIK3R6* and 0.920 for *SHOX2*, indicating excellent discriminative performance (Fig. 4A and B). Expression boxplots based on ED data demonstrated that these two genes were notably downregulated in the ED group in comparison to controls (Fig. 4C and D). Similarly, expression boxplots using PD data revealed significant upregulation of these two genes in the PD group (Fig. 4E and F). These findings suggested the potential of *PIK3R6* and *SHOX2* as key genes for screening and investigating molecular mechanisms underlying disease phenotypes, providing a foundation for exploring differential gene expression and associated biological features in basic research.

### Immunological infiltration analysis

**Fig. 5:**
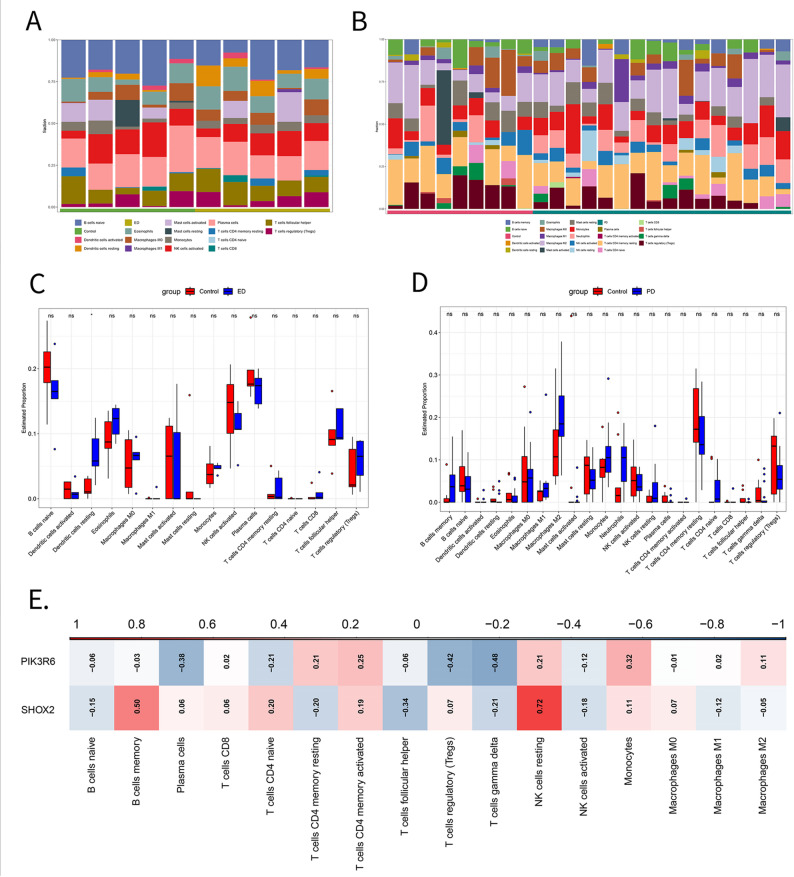
Immune infiltration analysis. (**A**) CIBERSORT expression matrix of ED and control samples. (**B**) CIBERSORT expression matrix of PD and control samples. (**C**) Boxplot showing the estimated proportion of different immune cell types in ED samples. (**D**) Boxplot illustrating the estimated proportion of different immune cell types in PD samples. (**E**) Heatmap of correlations between genes and immune cells. ED = Erectile Dysfunction, PD = Parkinson’s Disease

KEGG enrichment analysis suggested a potential correlation between immune responses and disease pathogenesis. Immune cell infiltration analysis for 22 immune cell types was performed to examine the potential immune responses associated with the DEGs. In the ED group, the proportion of resting dendritic cells (DCs) was significantly elevated compared to controls, as revealed by CIBERSORT analysis and corresponding boxplot visualization (Fig. 5A and C). In the PD group, memory B cells and M2 macrophages were more abundant in comparison to the controls (Fig. 5B and D).

Finally, immune correlation analysis was conducted on the two selected genes, and a heatmap illustrating gene-immune cell correlations was generated. The *PIK3R6* gene exhibited a strong negative correlation with regulatory T cells and gamma delta T lymphocytes, with correlation coefficients of −0.42 and − 0.48, respectively. This finding indicated that higher *PIK3R6* expression was linked to lower proportions of these T cells. Conversely, *SHOX2* was strongly positively correlated with the number of resting NK cells and memory B cells, with correlation coefficients of 0.72 and 0.50, respectively. This finding revealed that elevated *SHOX2* expression was related to higher proportions of these two cell types (Fig. 5E). The relationship between correlation coefficient and p-value is provided in Table 4.

**Table 4:** The relationship between correlation coefficient and p-value

Correlation Coefficient (*r*)	Strength of Correlation	*P*-value Range	Significance
0.8–1.0	Very Strong	< 0.001	***
0.6–0.8	Strong	0.001–0.01	**
0.4–0.6	Moderate	0.01–0.05	*
0.2–0.4	Weak	> 0.05	Not Significant
0.0–0.2	Very Weak	> 0.05	Not Significant

### PPI network and prediction of related miRNAs

**Fig. 6:**
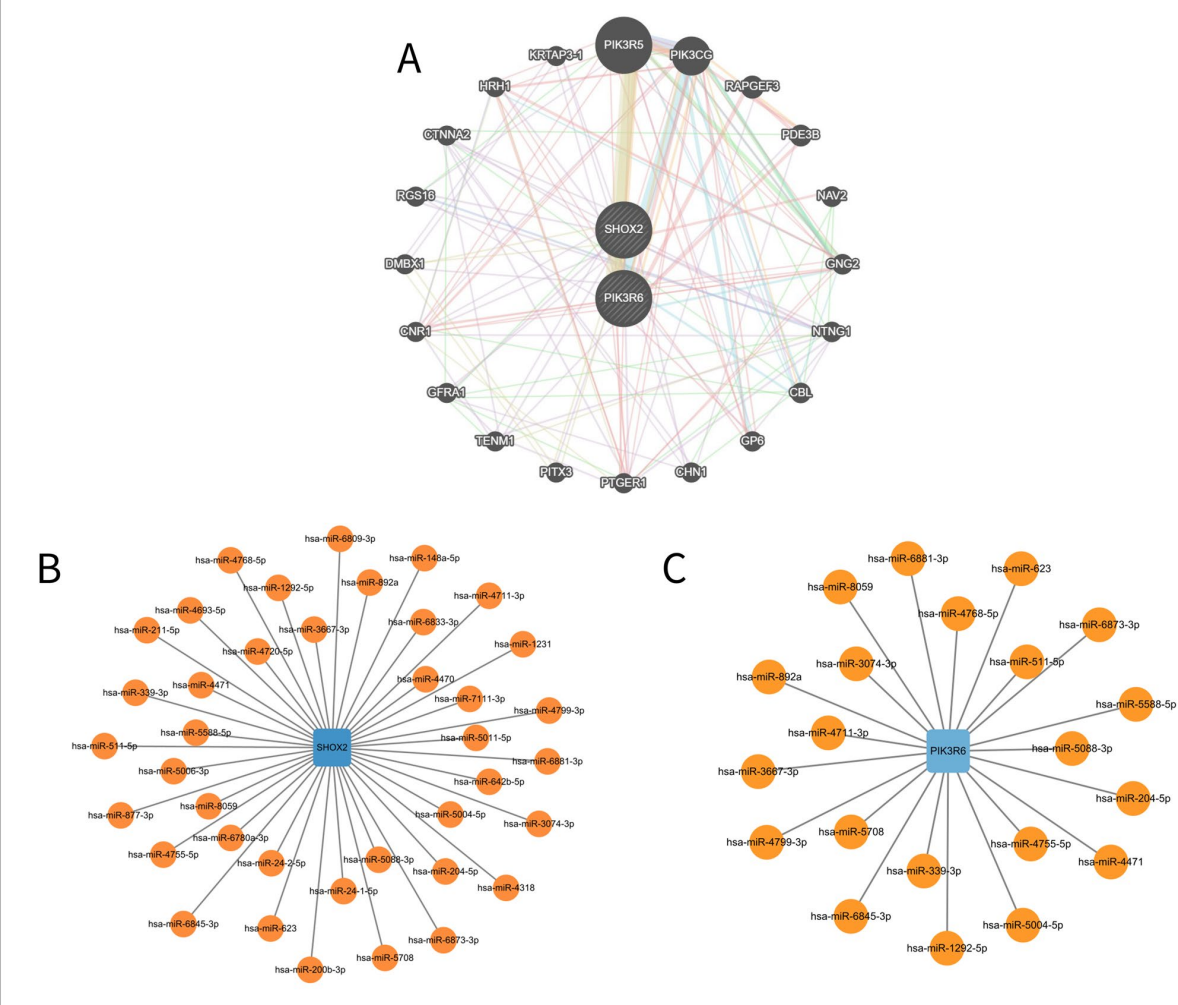
Gene-related and miRNA-related interaction network. (**A**) GeneMANIA gene interaction network. (**B**) and (**C**) miRNA interaction networks of the two key genes

To further investigate the screened genes, a GeneMANIA interaction network was established to illustrate complex gene interactions. Different colored lines represent various types of relationships between genes, including physical interactions, co-expression, and predicted associations (Fig. 6A). Additionally, miRNA interaction networks related to the two key genes were predicted (Fig. 6B and C), reflecting the relevance of post-transcriptional regulation. These analyses provide insights into the regulatory mechanisms involving *SHOX2* and *PIK3R6*.

### External validation of key candidate genes

**Fig. 7:**
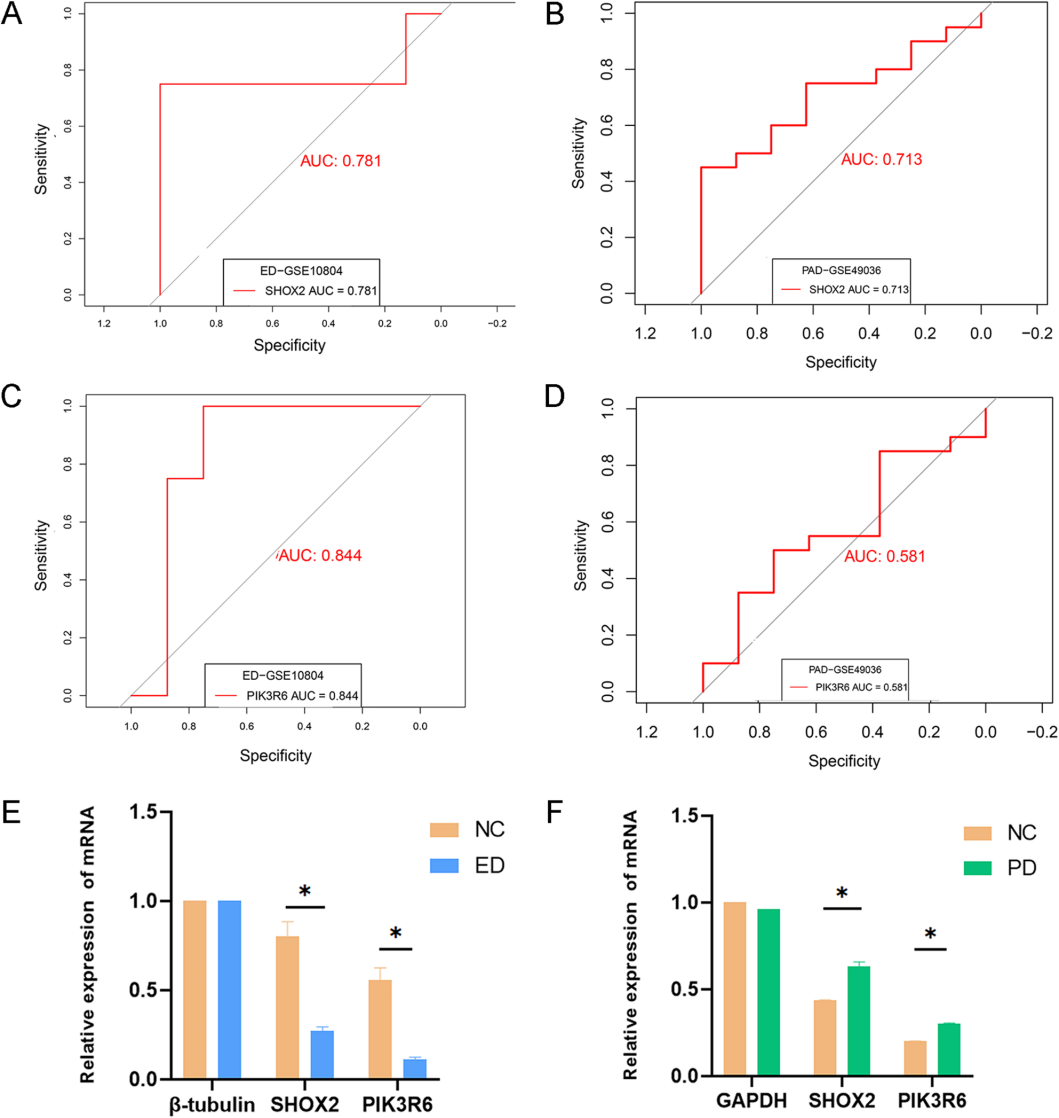
External and experimental validation. (**A**) and (**C**) ROC curves of DEGs in the validation dataset GSE10804. (**B**) and (**D**) ROC curves of DEGs in the validation dataset GSE49036. (**E**) mRNA expression levels of β-tubulin and *SHOX2*,* PIK3R6* in ED and control groups. (**F**) mRNA expression levels of β-tubulin and *SHOX2*,* PIK3R6* in PD and control groups.* indicates *P* < 0.05

External validation was performed using independent ED- and PD-related datasets to validate the significance of the shared genes. DEA was conducted in the GSE10804 and GSE49036 datasets, followed by ROC curve analysis. In the validation dataset GSE10804, the AUC value of *SHOX2* was 0.781, and that of *PIK3R6* was 0.844 (Fig. 7A and C). In the validation dataset GSE49036, the AUC value of *SHOX2* was 0.713, and that of *PIK3R6* was 0.581 (Fig. 7B and D). These results supported the good diagnostic performance of these two genes. The boxplots of two validation sets have been added to Supplementary Fig. 2.

### Experimental validation of key candidate genes

Cavernous tissue and midbrain tissue samples were collected from control and experimental model rats to experimentally verify the identified hub genes linking ED and PD. The expression profiles of *SHOX2* and *PIK3R6* in both groups were assessed via qRT-PCR. A significant downregulation of *SHOX2* and *PIK3R6* was found in the ED group in comparison to controls (*p* < 0.05). Additionally, the expression levels of both genes were lower than that of the reference gene β-tubulin (Fig. 7E). In the PD group, both *SHOX2* and *PIK3R6* genes were upregulated compared to the control group and were statistically significant (*p* < 0.05) (Fig. 7F).

## Discussion

ED is a common multifactorial disorder that may result from organic or psychological factors, substantially affecting the quality of life of affected men and their partners [25]. It is primarily marked by the consistent inability to achieve and maintain an erection sufficient for satisfactory sexual intercourse [1]. The primary features of PD encompass rigidity, tremor, bradykinesia, and postural instability. The disease is accompanied by various non-motor symptoms, among which autonomic dysfunction may contribute substantially to overall disability. ED is a common non-motor symptom observed in male patients with PD [9].

The sexual function needs of individuals with PD should not be overlooked [26]. Existing research demonstrates a greater prevalence of sexual dysfunction in individuals with PD, with ED being the most common type [27]. In recent years, the correlation between ED and PD has garnered growing research interest [28, 29]. However, studies investigating the genetic links between the two remain limited. By applying a comprehensive bioinformatics approach, this research analyzed the microarray data of ED and PD from the GEO datasets GSE2457 and GSE7621. The DEA identified 474 key genes in the ED dataset and 1148 module genes linked to PD. Significant differences in the gene expression patterns between disease and normal samples were found in the heatmaps and volcano plots. Ultimately, 25 hub genes were identified.

GO analysis demonstrated that the identified genes were primarily involved in such biological processes as pyruvate metabolism, sugar metabolism, and nucleotide metabolism. DM is a chronic metabolic disease marked by impaired glucose metabolism and increased blood glucose levels [30]. It is a common complication of ED, affecting about three-quarters of diabetic men [31]. KEGG enrichment analysis indicated that PD was linked to pathways such as viral infections and cytokine-cytokine receptor interactions. Notably, the potential link between viral exposure and the risk of neurodegenerative illnesses has been confirmed [32]. For instance, herpesvirus and cytomegalovirus infections may accelerate the degeneration of dopaminergic neurons through direct neurotoxicity and the promotion of inflammation, ultimately contributing to neurodegenerative changes in the CNS [33]. These results may offer a theoretical basis for further elucidating the roles of these genes in the pathogenesis of both ED and PD.

Key genes were further refined using ML algorithms, including LASSO regression and SVM-RFE. Their potential diagnostic performance was confirmed via ROC analysis. Immune infiltration analysis demonstrated that these key genes were correlated with multiple immune cell types, particularly mast cells, plasma cells, B cells, and DCs, highlighting the significance of immune regulation in the pathogenesis of ED and PD.

After screening, *SHOX2* and *PIK3R6* were identified as significant genes by ML and were also among the 25 DEGs. Previous studies reveal that *SHOX2* is closely related to cardiac pacing, skeletal development, and tumorigenesis. Currently, its potential involvement in PD is still unclear. This research demonstrated that *SHOX2* was significantly differentially expressed in PD patients. Although no direct evidence currently links *SHOX2* to PD, its biological functions and the pathological mechanisms of PD suggest that *SHOX2* may regulate the development or survival of dopaminergic neurons, similar to other transcription factors (such as *FOXA2*) in PD [34]. Additionally, *SHOX2* may affect oxidative stress or mitochondrial function pathways via epigenetic mechanisms and regulate protein degradation or inflammatory responses in conjunction with risk genes (such as *PIK3CA* and *LRRK2*), thereby contributing to PD-related metabolic abnormalities, including elevated levels of 8-hydroxy-2’-deoxyguanosine [35]. Further functional experiments are warranted to elucidate the precise role of *SHOX2* in PD and to assess its potential as an early diagnostic marker. *PIK3R6*, a member of the phosphoinositide 3-kinase (*PI3K*) family, is a crucial regulatory subunit of the *PI3K/Akt* signaling pathway. It may indirectly participate in the pathogenesis of ED by regulating vascular endothelial function. Existing studies demonstrate that the *PI3K/Akt* pathway is essential for maintaining endothelial cell function, such as promoting the synthesis of nitric oxide (NO) through activation of endothelial nitric oxide synthase (*eNOS*), with NO acting as a crucial mediator of penile erection [36]. Abnormal expression of *PIK3R6* can disrupt *PI3K/Akt* signaling, resulting in endothelial cell dysfunction and impaired relaxation of cavernous smooth muscle and ultimately causing ED. Moreover, the interplay between the *PI3K/Akt* pathway and metabolic diseases (such as hyperlipidemia) may further aggravate ED progression. Hyperlipidemia independently contributes to the risk of ED. It can induce oxidative stress and inflammatory responses, inhibit *PI3K/Akt/eNOS* activity, decrease NO bioavailability, and promote vascular endothelial dysfunction, thereby impairing erectile function [37]. Notably, clinical studies have confirmed that the prevalence of ED is notably increased among individuals with hyperlipidemia, and metabolic syndrome related to lipid abnormalities represents an independent risk factor for ED [38]. Therefore, *PIK3R6* may serve as a molecular link between hyperlipidemia and ED through regulation of the *PI3K/Akt* pathway, potentially explaining the association between gene expression differences and ED phenotypes. Further experimental research is required to validate the specific function of *PIK3R6* in ED models and its regulatory interaction with the *PI3K/Akt* pathway. ML analyses identified *SHOX2* and *PIK3R6* as key genes, suggesting their potential as effective targets. Furthermore, this study deeply interpreted regulatory networks. We not only predicted miRNAs that interacted with *SHOX2* and *PIK3R6*, but also found through literature review that certain miRNAs, such as miR-200a-3p, were associated with ED and PD. In ED, it has been proved that miR-200a can alleviate erectile dysfunction in diabetes rats by regulating phenotype conversion, apoptosis and fibrosis [39]. In PD, its abnormal expression is associated with neuronal apoptosis and oxidative stress [40]. Our research predicts that it may interact with *SHOX2* or *PIK3R6*, even though it is a regulatory mechanism. We speculate that oxidative stress is a core pathological process shared by ED and PD. Nevertheless, the reliance on public datasets may introduce inherent biases in our findings. Thus, further experimental studies and analyses of larger, independent patient cohorts were needed to verify these results.

Furthermore, verification of these genes will be conducted in the future by assessing their expression levels in animal models. This step is crucial to confirm the differential expression of genes under disease conditions and to elucidate their specific roles in disease onset and progression. Validating these genes in animal models will enable a more accurate evaluation of their relevant potential for ED and PD. This process will deepen understanding of gene functions.

Due to the specificity of the diseases, the sample sizes and quality in the database are limited. Firstly, the specific biological mechanisms underlying the DEGs remain inadequately elucidated.Secondly, the human datasets used for analysis lacked sample-level clinical metadata such as age and gender, and thus our model was not adjusted for these potential confounding factors. In addition, this study was limited to rat models with diabetes-induced ED. Future research is necessary to detect the expression patterns of these two key genes in other classic ED models (such as cavernous nerve injury models, old animal models) or in human patient cohorts classified according to different etiologies, so as to confirm whether they are broad-spectrum biomarkers for ED. Thirdly, our external validation strategy was inconsistent. The initial finding was derived from a rat model with diabetes, whereas the external validation of ED was performed using a human endothelial cell dataset (GSE10804). Although this study provides preliminary supportive evidence, our findings need to be validated in more biologically matched systems. Hence, further experimental validations and prospective studies are required. In addition, psychological and social factors play important roles in the progression of ED and PD. A substantial proportion of ED patients are psychogenic rather than organic [41]. PD is frequently accompanied by mental health issues, with studies indicating a higher incidence of such problems in PD patients compared to the general population [42]. Given the genetic focus of this study, psychological and social factors were insufficiently addressed. Therefore, subsequent research will be conducted to investigate the shared molecular mechanisms of ED and PD.

## Conclusions

This research, through multi-omics analysis, reveals for the first time the shared biological functions and potential common DEGs between ED and PD. Key hub genes, including *SHOX2* and *PIK3R6*, were identified and validated using the online database. These findings might offer new insights, potential biomarkers for future research.

## Supplementary Information

Supplementary Material 1

## Data Availability

Data are available at NCBI GEO: GSE2457, GSE7621, GSE10804, GSE49036.

## Abbreviations

ASRGs: Androgen-sensitive related genes
AUC: Area under the curve
cDNA: Complementary DNA
CNS: Central nervous system (
DEGs: Differentially expressed genes
ED: Erectile dysfunction
FEA: Functional Enrichment Analysis
GEO: Gene Expression Omnibus
GEPs: Gene expression profiles
GO: Gene Ontology
KEGG: Kyoto Encyclopedia of Genes and Genomes
LASSO: Least Absolute Shrinkage and Selection Operator
PD: Parkinson’s disease
ROC: Receiver operating characteristic
SE: Standard error
STZ: Streptozotocin
SVM-RFE: Support Vector Machine Recursive Feature Elimination
