# Supplementary material for: Identification and validation of shared key genes between Parkinson’s disease and erectile dysfunction: a bioinformatics approach
Source: Hereditas. 2025 Nov 25;163:3. doi: 10.1186/s41065-025-00614-1 (PMC12764067; doi:10.1186/s41065-025-00614-1)
Supplement: Supplementary file 1 — Supplementary Material 1 [file 41065_2025_614_MOESM1_ESM.docx]

**Supplementary Materials**

**
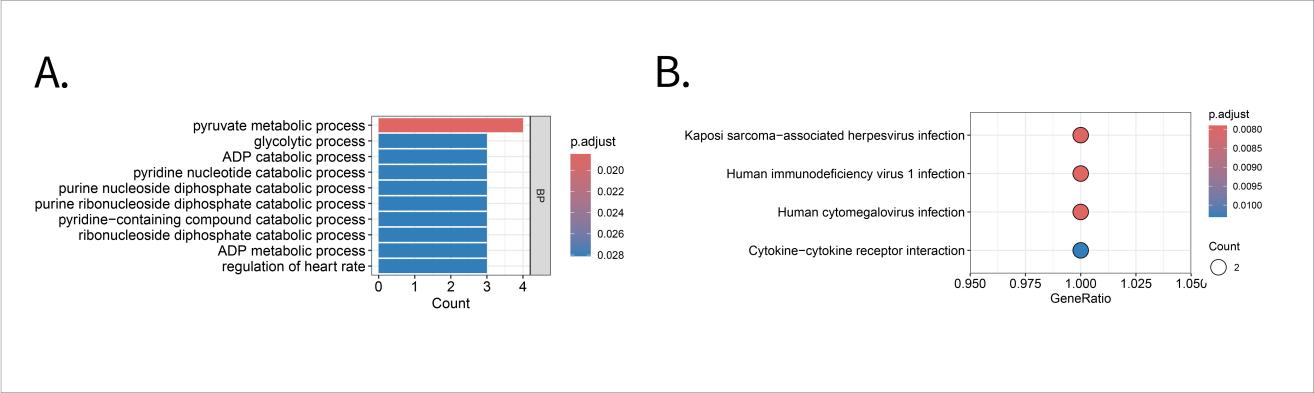
**

**Supplementary Figure 1** Intersection gene KEGG and GO analysis results

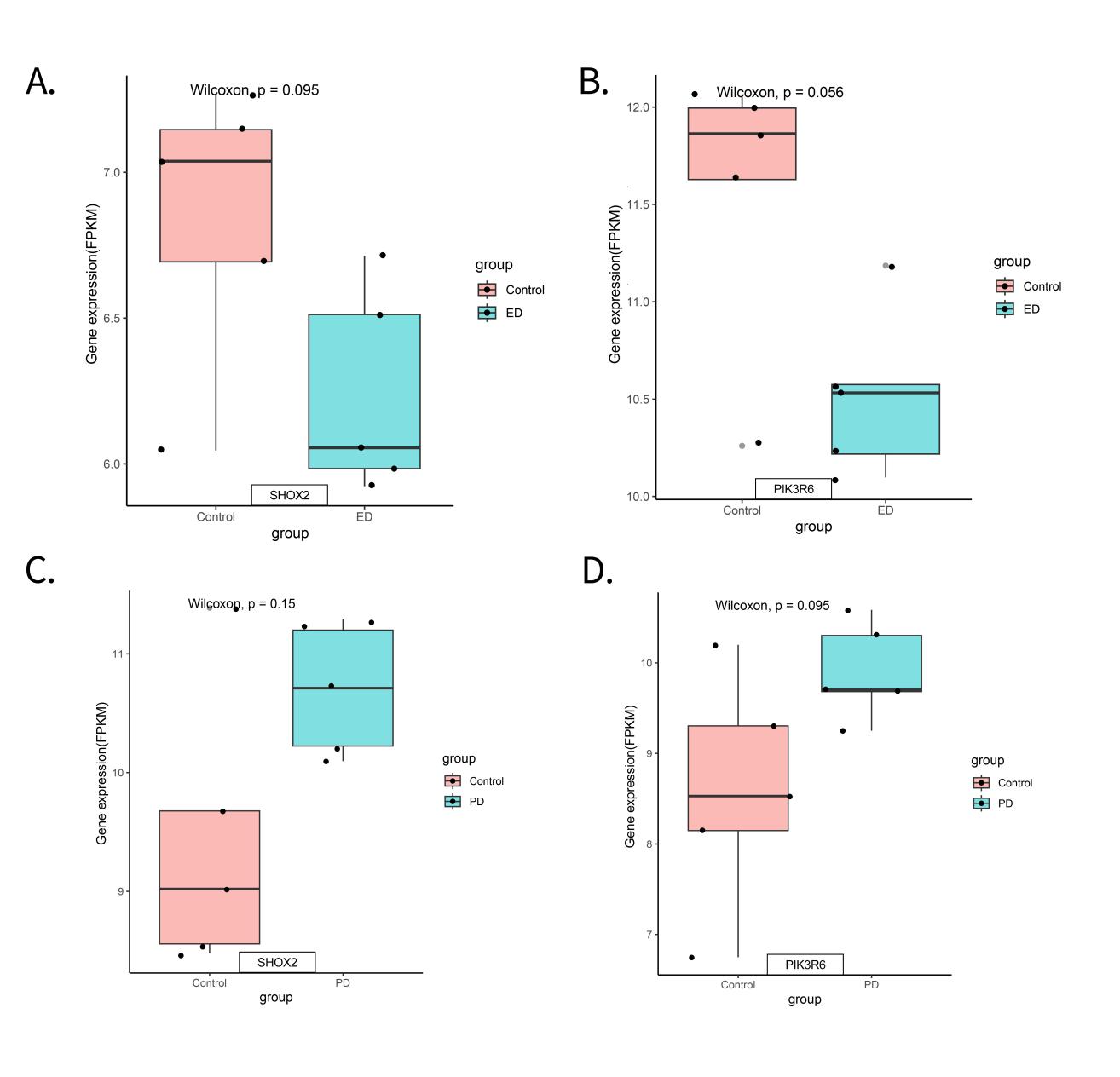


**Supplementary Figure 2** Box expression diagrams of two validation sets
